# Spatial Analyses of Specialized Metabolites: The Key to Studying Function in Hosts

**DOI:** 10.1128/mSystems.00148-17

**Published:** 2018-03-06

**Authors:** Melissa M. Galey, Laura M. Sanchez

**Affiliations:** a Department of Medicinal Chemistry and Pharmacognosy, University of Illinois at Chicago, Chicago, Illinois, USA

**Keywords:** chemical communication, imaging mass spectrometry, specialized metabolites

## Abstract

Microbial communities contribute to a wide variety of biological functions in hosts and have the ability to specifically influence the health of those organisms through production of specialized metabolites. However, the structures or molecular mechanisms related to health or disease in host-microbe interactions represent a knowledge gap. In order to close this gap, we propose that a combinatory approach, pulling from microbiology and analytical chemistry, be considered to investigate these interactions so as to gain a better understanding of the chemistry being produced. We hypothesize that bacteria alter their chemistry in order to survive and induce specific states in their host organisms. Our lab makes use of imaging mass spectrometry and other analytical techniques to study this chemistry *in situ*, which provides actionable information to test hypotheses.

## Abstract (precis)

Microbial communities contribute to a wide variety of biological functions in hosts and have the ability to specifically influence the health of those organisms through production of specialized metabolites. However, the structures or molecular mechanisms related to health or disease in host-microbe interactions represent a knowledge gap.

## PERSPECTIVE

To date, microbiome studies have demonstrated that microbial communities have the ability to significantly influence the health of their host organisms through the production of specialized metabolites (SMs) (1). Many studies have revealed that the presence of these microbes directly correlates with the presence of specific metabolites from discrete locations through the use of tandem mass spectrometry and molecular networking (2, 3). While these studies investigate the presence of bacteria in or on a host, they do not explicitly elucidate the molecular mechanisms and SMs produced by the microbes that may influence a host organism’s health. Therefore, to move forward in our knowledge of the impact that microbial communities have on host health, it is imperative that model systems and tools be developed to better study these interactions *in situ*. We believe that it is increasingly important to work toward building a comprehensive inventory of the SMs bacteria utilize for survival and infection within a host to test and design better treatments for disease. Previous studies have focused on the introduction of specific microbial species or whole communities to gnotobiotic animal models in order to determine the full effect that these microorganisms have on their hosts, whether it be influencing a disease state or the overall microbiome of the host (4, 5). While these types of animal studies have proven to be enlightening in terms of establishing the influence a specific microbe(s) or whole communities have on host organisms, it can fail to take into account the interactions found between native microbial species normally found within the host. A multitude of processes within a gnotobiotic host would not be observed, such as bile acid recycling in the enterohepatic system, due to the fact that specific microbial community members may not be present (6). This leads to an incomplete understanding of the chemical communication involved in host-microbe interactions, as the microbial origins of SMs being produced are still unknown. In addition to using gnotobiotic animal models to study microbe-host interactions, researchers have also taken an interest in the interactions between host tissue and its inherent microbiota. In a recent paper by Ismail et al., a unique relationship is observed between mammalian epithelial cells and enteric quorum-sensing bacteria, where *Vibrio harveyi* is used as the model bacterial species (7). The research findings point to the existence of a quorum-sensing mimic produced by the mammalian cells which aids in communication between the host and its bacterial partners. While this type of information has an exciting potential to realize host-microbe communication, the underlying chemistry remains elusive; this has limited further studies or an expansion to other host-microbe systems. In order to better study these interactions along with the functional role of bacteria in health and disease, it is necessary to establish analytical methodologies and tools to fully explain the interchange of SMs from bacteria to their hosts.

**FIG 1:**
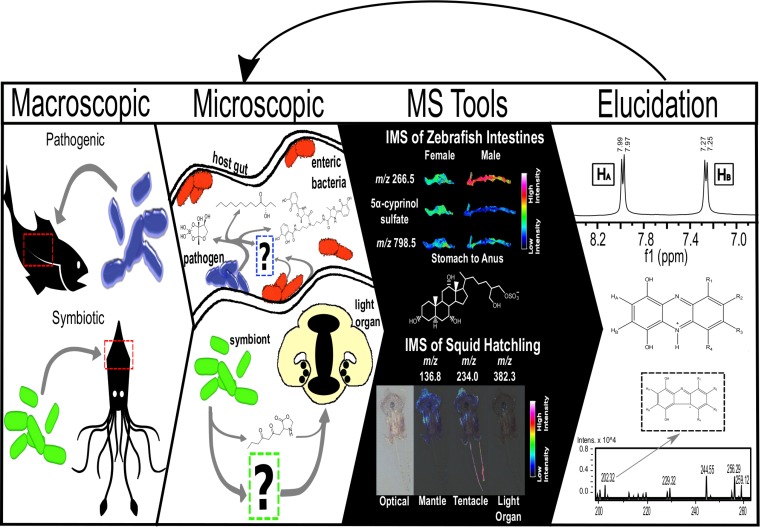
Pathogenic and symbiotic bacteria have intimate relationships with their hosts resulting in complex chemical communication. We propose this workflow to identify and elucidate the molecular mechanisms and structures produced in host-microbe interactions. This will allow us to connect the presence and production of SMs to their respective gene clusters in the microbial genome. We first investigate these interactions at a macroscopic level and then observe them at a microscopic level using IMS to examine tissue samples. This technique lends itself to high spatial resolution analyses for untargeted metabolomics; however, it is also disadvantageous because it is destructive to the sample, which can make it difficult to integrate it with other types of analyses such as microbial sequencing. A possible way to overcome this would be to designate every other tissue slice for different analysis techniques or require biological replicates. Industrial and academic labs are actively creating software that can incorporate statistical analyses for IMS data sets, such as SCiLs. This allows for the easy identification of exogenous metabolites in host samples, such as is the case of 5α-cyprinol sulfate, a bile salt originating from fish intestines. This information will then enable us to better connect a genotype with an observed chemotype and phenotype within a system, leading to a better understanding of chemical communication in host-microbe interactions. The IMS data sets of the zebrafish intestines and squid hatchling were obtained using spatial resolutions of 50 μm and 20 μm, respectively. Both samples used a 50:50 (wt/wt) mixture of α-cyano-4-hydroxycinnamic acid and 2,5-dihydroxybenzoic acid as a matrix.

As we move toward closing the knowledge gap that lies in how microbial communities specifically impact their host, whether as a pathogen or symbiont, we advocate that a combinatory approach that includes both microbiology and analytical chemistry techniques should be employed to probe these interactions in the next 5 years. In our own lab, we hypothesize that bacteria alter their chemistry in order to survive and induce specific states in their host organisms. Therefore, it is imperative to study this microbial chemistry *in situ* to elucidate how these chemical communication networks function within organisms. In particular, it is of great interest to understand how microbial communities interact with host tissues at different stages of colonization and expansion to learn more about how they thrive in their host environment. To accomplish this goal, the scientific community must develop tools that cannot only better model disease states but also allow for *in situ* chemical dialogue that occurs as a result of the bacterium-host interaction. In the Sanchez lab, we incorporate matrix-assisted laser desorption ionization–time of flight mass spectrometry (MALDI-TOF MS), which is advantageous for use in the study and elucidation of SMs. This analytical technique is solvent-free and highly sensitive and also has a large dynamic range, which allows for the detection of a high number of SMs at a wide range of mass-to-charge (*m*/*z*) ratios. Additionally, we make use of imaging mass spectrometry (IMS), which allows for the introduction of a two-dimensional spatial component to the study of host microbiomes. The application of IMS also results in increased visualization of the true spatial distribution of SMs in/on a sample, over other practiced methods of molecular cartography. Our lab is actively exploring the chemical dialogue found in the host-microbe interactions by utilizing a combination of microbiology and innovative mass spectrometry techniques, such as IMS, molecular networking, and structure elucidation, to identify chemistry in host systems. This is best illustrated by Fig. 1, which outlines our approach to better understand these complex relationships. In order to best probe microbe-host interactions, our research has focused on two model systems, which consist of both pathogenic and symbiotic relationships.

Further investigation of a pathogenic interaction led to the initiation of one of our current projects involving *Danio rerio* and *Vibrio cholerae*. It has been demonstrated that zebrafish are a putative zoonotic reservoir for *V. cholerae* and closely mimic the human disease, cholera (8). Zebrafish contract the disease upon waterborne exposure and subsequently transmit the bacterium to naive fish. Our work initiates infection of transparent Casper (9) zebrafish with fluorescently tagged *V. cholerae* to visualize infection and clearance from the fish digestive tract. Following infection and colonization, our lab makes use of IMS to observe spatial distribution of small molecules produced by *V. cholerae* and its host as well as any other compounds produced by enteric microbiota in response to the presence of a pathogen. Since IMS allows for the examination of spatial distributions of SMs throughout the intestinal tissue or the host organism, we can also use this technique to observe and identify metabolites that are differentially regulated in response to the presence of *V. cholerae*. This will allow our lab to better characterize the interaction between *V. cholerae* and its host organism, as we will have a better understanding of the chemical communication responsible for colonization and expansion. As a future result of this research, we may be able to augment our drug discovery capabilities due to the fact that we can elucidate SMs that are produced as a result of host-microbe interactions.

While host colonization by a bacterium has often been thought of as an unwanted invader that brings disease, there are some situations in which these colonizations bring about a symbiotic, or mutually beneficial, relationship such as in the case of *Euprymna scolopes* and *Vibrio fischeri*. Shortly after birth, the light organ of the Hawaiian bobtail squid is colonized by symbiotic communities of *V. fischeri* (10). This allows the host to take advantage of the bioluminescence produced by its microbial inhabitants for counterillumination to hide from both predators and prey in the ocean (11). In collaboration with the lab of Mark Mandel at the University of Wisconsin—Madison, we can assess the colonization potential of different *V. fischeri* mutants and transposons (12). Our lab is optimizing and conducting IMS on squid hatchlings inoculated with *V. fischeri* strains to determine differences in the chemistry produced between strains and the spatial distribution of the molecules produced within the host tissue. Additionally, we are in the process of characterizing these bacterial strains using our novel MALDI-TOF MS-based workflow that allows for rapid proteomic and metabolomic classification (13). We hope to gain a better understanding of the symbiotic relationship between the squid and *V. fischeri* and how colonization in this model system may translate to similar host-microbe interactions.

The combination of microbiology and mass spectrometry will allow us to better probe and identify SMs produced during colonization and to investigate conserved chemistries from bacteria in the same genus that have similar colonization potentials. Using tandem mass spectrometry allows us to observe similarities and differences among strains in one genus (14, 15). Therefore, we can explore colonization and expansions from a metabolomic (chemical) lens.

Moving forward, we envision more interdisciplinary work between SM chemists, biologists, and computer scientists to elucidate chemical pathways within a system. In order to accomplish this goal, collaborative teams will work together to create workspaces that rely on existing workflows that are already routinely used by laboratories with the added benefit that members of the geographically distant labs will be able to reanalyze or explore data sets that each lab has made available. A significant benefit of designing computational pipelines and making the results freely available to the community or other interested collaborators would be to increase transparency in the data and conclusions by removing subjectivity and providing provenance of conclusions. We have previous knowledge of some of the molecules that bacteria produce when grown axenically, but culturing *in vivo* (i.e., in a natural host) has the potential to unlock a plethora of new chemistry that is waiting to be discovered! By working together across different fields, we can combine our skill sets to better understand the molecules that drive both disease and symbiosis within a host-microbe interaction and move on, determining causation as opposed to remaining in a correlative stage. As shown in Fig. 1, we hope to better connect a genotype to a specific chemo- and phenotype, which will then allow us to better design and test hypotheses for the contributions from either the host or the microbe. We envision that in the next 5 years we can then use this chemistry to answer some of nature’s more challenging questions involving microbial chemistry and determining its functional role in the host-microbe environment.
